# Baicalein Inhibits *Staphylococcus aureus* Biofilm Formation and the Quorum Sensing System *In Vitro*

**DOI:** 10.1371/journal.pone.0153468

**Published:** 2016-04-29

**Authors:** Yan Chen, Tangjuan Liu, Ke Wang, Changchun Hou, Shuangqi Cai, Yingying Huang, Zhongye Du, Hong Huang, Jinliang Kong, Yiqiang Chen

**Affiliations:** The Institute of Respiratory Diseases, First Affiliated Hospital, Guangxi Medical University, Nanning, China; Université d'Auvergne Clermont 1, FRANCE

## Abstract

Biofilm formed by *Staphylococcus aureus* significantly enhances antibiotic resistance by inhibiting the penetration of antibiotics, resulting in an increasingly serious situation. This study aimed to assess whether baicalein can prevent *Staphylococcus aureus* biofilm formation and whether it may have synergistic bactericidal effects with antibiotics *in vitro*. To do this, we used a clinically isolated strain of *Staphylococcus aureus* 17546 (t037) for biofilm formation. Virulence factors were detected following treatment with baicalein, and the molecular mechanism of its antibiofilm activity was studied. Plate counting, crystal violet staining, and fluorescence microscopy revealed that 32 μg/mL and 64 μg/mL baicalein clearly inhibited 3- and 7-day biofilm formation *in vitro*. Moreover, colony forming unit count, confocal laser scanning microscopy, and scanning electron microscopy showed that vancomycin (VCM) and baicalein generally enhanced destruction of biofilms, while VCM alone did not. Western blotting and real-time quantitative polymerase chain reaction analyses (RTQ-PCR) confirmed that baicalein treatment reduced staphylococcal enterotoxin A (SEA) and α-hemolysin (*hla*) levels. Most strikingly, real-time qualitative polymerase chain reaction data demonstrated that 32 μg/mL and 64 μg/mL baicalein downregulated the quorum-sensing system regulators *agrA*, RNAIII, and *sarA*, and gene expression of *ica*, but 16 μg/mL baicalein had no effect. In summary, baicalein inhibited *Staphylococcus aureus* biofilm formation, destroyed biofilms, increased the permeability of vancomycin, reduced the production of staphylococcal enterotoxin A and α-hemolysin, and inhibited the quorum sensing system. These results support baicalein as a novel drug candidate and an effective treatment strategy for *Staphylococcus aureus* biofilm-associated infections.

## Introduction

Like many other pathogens, *Staphylococcus aureus* tends to form biofilms on medical implants or damaged tissue [1,2], rather than living as free planktonic cells within the host. This is concerning because *S*. *aureus* biofilms on implanted devices and catheters commonly cause chronic and persistent infections. Indeed, the increasing resistance of *S*. *aureus* biofilms to multiple drugs has become a major threat to human health.

To form biofilms, gram-positive bacteria use oligopeptides as signaling molecules, the universal language for intraspecific communication [3]. This quorum sensing (QS) system is a potential target for the treatment of bacterial biofilm infections. In fact, researchers using an automated docking program to screen traditional Chinese Medicines (TCMs) have identified several QS inhibitors [4]. Our prophase studies [5] showed that *Scutellaria baicalensis* liquid (a TCM) can inhibits *S*. *aureus* biofilm formation. Baicalein (an extract of *Scutellaria baicalensis*) possesses antibacterial activity, displays low toxicity in humans, and has been used for more than a thousand years in China. Thus, baicalein has the potential to be an effective treatment strategy against *S*. *aureus* biofilm-associated infections.

We aimed to determine whether baicalein could effectively prevent *S*.*aureus* biofilm formation to a degree that could be correlated with increased antibiotic susceptibility. In addition, we endeavored ascertain whether baicalein could interfere with the QS system and affect bacterial virulence.

## Materials and Methods

### Bacterial strain and *in vitro* antibacterial treatment

#### Bacterial strain and the culture medium

Of the 21 clinical *S*. *aureus* strains separated in our preliminary work, the isolate *S*. *aureus* 17546 (t037) was chosen because it has the strongest ability to form biofilms. It was the stole strain studied in the experiments discussed below. The standard strain *S*. *aureus* ATCC 29213 was used for quality control.

We grew the *S*.*aureus* 17546 (t037) in tryptic soy broth supplemented with 0.5% glucose (TSB-G) medium for biofilm formation using a polystyrene carrier (10×10 mm^2^) placed in 24-well plates (JET BIOFIL). The bacterial suspension was diluted by TSB-G to an absorbance of OD_600_ = 0.1 for use in the rest of the remainder of the study.

#### Antimicrobial treatment

Samples of baicalein (Sigma) were tested using high-performance liquid chromatography to confirm purity at > 98%. They were then dissolved in dimethyl sulfoxide (DMSO) and stored at -70°C until used. The pH of the solutions was controlled at 7.2–7.4.

To determine if sub-inhibitory concentrations of baicalein would eradicate *S*. *aureus*, baicalein was added to the bacterial broth (1000 colony forming units; CFUs) at final sub-inhibitory concentrations of 64, 32, and 16 μg/mL and DMSO concentrations of 0.8%, 0.4%, and 0.2%, respectively (note that the minimum inhibitory concentration of DMSO is 6.4%). For comparison, clarithromycin (CLR) and vancomycin (VCM; Sigma) were added to the bacterial broth to final concentrations of 16 and 4 μg/mL, respectively. Next, the minimum inhibitory concentrations (MICs) of baicalein, CLR, and VCM were determined using a broth microdilution method according to the guidelines set by the Clinical and Laboratory Standards Institute (CLSI, 2012). Finally, the effects of 64, 32, and 16 μg/mL baicalein on the growth of *S*. *aureus* were measured over 26 h in terms of absorbance (optical density measured at a wavelength of 600 nm; OD600).

### Inhibition *in vitro*

#### Biofilm bacterial CFU counts

*Staphylococcus aureus* 17546 bacteria (separated from the clinical microbiology inspection center of first affiliated hospital, Guangxi Medical University, China) were grown overnight in TSB-G. The next day, it was and diluted with TSB-G to an absorbance of OD_600_ = 0.1. Then, 2 mL of the bacterial suspension was applied to sterile polystyrene 24-well plates with baicalein (final concentrations of 64, 32, and 16 μg/mL), noting that the DMSO in each plate was lower than its MIC. Bacteria were grown at 37°C without shaking, and the medium (TSB-G and baicalein) was refreshed every other day for 3 and 7 days to form biofilms. After washing and sonication to disrupt clumps, the biofilm bacterial CFU counts were determined by plating serial dilutions on Luria broth (LB) agar. The experiments were performed three times in parallel.

#### Crystal violet staining

Two different crystal violet staining experiments were performed. First, to determine whether baicalein could inhibit biofilm formation, we clinically separated several of the 21 confirmed *S*. *aureus* strains (17413, 17546, 17624, 18558, 18565, and 18791) and treated them with 32 μg/mL baicalein for 3 days. The strains separated by resistant spectrum of sub-type and protein A (spa) sub-type. Then, crystal violet staining was performed and the optical density measured at a wavelength of 595 nm (OD_595_).

For the second experiment, unbound cells from the *S*. *aureus* 17546 (t037) 3- and 7-day incubation plates were removed by washing twice with phosphate-buffered saline (PBS). The biomass of each slice was air-dried and then stained for 20 min with filtered 0.5% crystal violet, washed three times with PBS to remove the unbound stain, dried, and solubilized in 2 mL of 95% ethanol for 3 min. Then, 200 μL of each solution was added to a 96-well plate and read at OD_595nm_ in an enzyme-linked immunosorbent assay microplate reader (Multiskan, Thermo Scientific). The experiments were performed for three times in parallel.

#### Fluorescence microscopy protocol

After treatment with 64, 32, and 16 μg/mL baicalein for 3 and 7 days, the biofilms of each group were stained with dyes (SYTO9^®^ BacLight^™^) for 15 min and then washed with PBS to remove the stains. Afterwards, we obtained fluorescent images using fluorescence microscopy (Olympus, CKX41).

### Combined efficacy *in vitro*

#### Biofilm bacterial CFU counts

To compare the synergistic effects of baicalein on *S*. *aureus* biofilms to the effects of VCM, 3- and 7-day biofilms were prepared in 24-well plates containing a polystyrene slice in the bottom of each well. The preparation of the biofilms was performed as described above. The slices were gently washed twice with PBS. Then, for 3-day assay, baicalein or CLR with or without VCM were added to each well in TSB-G for a total volume of 2 mL per well. The final concentrations of baicalein, CLR and VCM were 32, 16, and 4 μg/mL. CLR was used here as a positive control [5]. Serial dilutions of baicalein with or without VCM were used for the 7-day biofilms. The final concentrations of baicalein were 64, 32, and 16 μg/mL and VCM concentration was aslo 4 μg/mL. Finally, the slices with biofilms were placed at the bottom of the wells again and cultivated at 37°C for 12 h. After the treatment was complete, the slices were washed with PBS to remove planktonic cells and sonicated to disrupt the clumps. The biofilm bacterial CFU counts were conducted by plating serial dilutions on LB agar. This experiment was performed three times in parallel.

#### CLSM protocol

We used confocal laser scanning microscopy (CLSM) to investigate whether the combination of baicalein plus VCM for 12 h could eradica *S*. *aureus* biofilms. Fluorescent LIVE/DEAD^®^ BacLight^™^ bacterial viability kit L7007 (Molecular Probes, Invitrogen) was used to detect live and dead cells according to the manufacturer’s instructions. Briefly, a 1:1 combination of fluorescent green (SYTO 9) and fluorescent red (propidium iodide) was used, as recommended in the product information sheet supplied by the manufacturer, to prepare a stained suspension in PBS. Bacteria with intact cell membranes would stain a fluorescent green, whereas those with damaged cell membranes would stain fluorescent red. Afterwards, the baicalein-treated biofilms (with and without 12 h of VCM) were incubated in a solution of 5 μL of the dye mixture and 5 mL of PBS at room temperature for 15 min in the dark. The slices were then rinsed three times with PBS and examined under CLSM (Nikon A1).

#### SEM protocol

Synergistic bactericidal effects were observed using a S3400N scanning electron microscope (SEM;Hitachi, Japan). For this experiment, we used the 3- and 7-day baicalein-treated or CLR-treated biofilms (with and without 12 h VCM). Each biofilm carrier was washed with PBS, fixed at room temperature with 2.5% glutaraldehyde for 24 h, and then rinsed three times in fresh PBS (pH 7.4). The carriers were passed through an ethanol gradient (e.g., 50%, 70%, 80% and 90%) for 15 min each, passed through 100% ethanol (three times for 10 min) for dehydration, dried, then coated with gold [5].

### Virulence factor assays

#### The extraction of toxins

Following rejuvenation, a single *S*. *aureus* colony was transferred to 3 mL TSB-G and cultured at 250 rpm and 37°C for the night. The next day, a 1/100 dilution of the culture was made in TSB-G and incubation continued. When the diluted culture reached OD_600_ = 0.3, a quarter of the suspension was supplemented with baicalein to final concentrations of 0, 16, 32 and 64 μg/mL. After an additional 5 h of incubation, each culture (100μL) was centrifuged at 12 000 rpm for 5 min, and each supernatant was filtered using a filter membrane with a diameter of 0.22 μm and reserved in the tubes for the next assays. At the same time, the bacterial count for each 100μL broth solution was determined by colony counting on LB agar. This process was performed three times.

#### Detection of Staphylococcal enterotoxin A

We used a western blotting assay technique developed by Rasooly [6] to detect staphylococcal enterotoxin A (SEA). Samples containing 100μL of supernatant were heated at 90°C for 5 min and immediately electrophoresed in a Mini-Protean II vertical dual-cell apparatus (Bio-Rad). The SEA bands were found by running discontinuous sodium dodecyl sulfate polyacrylamide gel electrophoresis [7] at a constant voltage (110 V) for 2 h. The protein band was visualized by staining with Coomassie brilliant blue R (Sigma). After 2 h of electrophoresis, the gel containing SEA (indicated by the marker) was cut down and dipped into Towbin transfer buffer, and the proteins transferred onto nitrocellulose membranes (Pall Corporation, USA) and blocked (5% nonfat milk in TBS with 0.1% Tween 20) for 60 min at room temperature. The gel was exposed to rabbit polyclonal antibodies (Sigma) and gently shaken for 1 h, then stored at 4°C overnight. Afterwards, it was washed with running buffer for 10 min and repeated three times. Next, the gel was exposed to SEA antibodies (Sigma) for 1 h at room temperature and washed three times. Bound antibodies were detected using an enhanced chemiluminescence western blot substrate (Pierce). Bands were visualized on X-ray films, which were then scanned and the images subsequently analyzed using Quantity One (Bio-Rad) to quantify the integrated density (pixels) of the bands. We calculated the amount of SEA in each cell by dividing each band by the logarithmic number of cells. The experiment was performed three times.

#### α-Hemolysin

To test *hla* production, 1 mL of *S*. *aureus–*baicalein supernatant was removed from from each group after centrifugation. The RNA was isolated and its gene expression detected through real-time quantitative polymerase chain reaction (RTQ-PCR) analysis. The following primers were used to amplify the 105 base pair (bp) sequence: 5’- ATG GCT CTA TGA AAG CAG CAG A -3’ (forward), and 5’- AAG GT GAA AAC CCT GAA GA -3’ (reverse). For the 83 bp 16S rRNA (the internal control housekeeping gene) sequence, the primers were 5’-CCA TAA AGT TGT TCT CAG TT -3’ (forward), and 5’-CAT GTC GAT CTA CGA TTA CT-3’ (reverse). The procedure for RNA isolation, cDNA synthesis, and RTQ-PCR is detailed in the following section. This experiment was performed three times in parallel.

### The expression of QS system-associated genes as detected by RTQ-PCR

#### Bacterial strains

We cultured the *S*. *aureus* strain on tryptic soy agar. A single colony was picked and inoculated into TSB-G (10 mL), followed by overnight agitation at 37°C. The following day, a 1/100 dilution of the culture was made in TSB-G (80 mL). Then, an aliquot (20 mL) of diluted suspension was added with baicalein to final concentrations of 0, 16, 32, and 64 μg/mL, with each sample containing the same concentration of DMSO (lower than the MIC;6.4%). These cultures were incubated at 37°C on an orbital shaker (180 rpm). A sample (1 mL) from each group was removed at 5 h, 24 h, and 72 h post-inoculation for RNA extraction and determination of OD_600_.

#### RNA isolation and analysis

We isolated RNA using a modification of the method developed by Sabersheikh et al [8]. The bacterial culture (1mL) was spun at 4000 rpm for one minute. The pellet was then suspended in a 100 μL mixture of lysostaphin (recombinant; 1 mg/mL; Sigma) and lysozyme (25 mg/mL; Sigma) and incubated at 37°C for 1 h. Next, it was centrifuged at 4000 rpm for 15 min, then lysed by addition of 1 mL TriPure Isolation Reagent (Roche) and homogenized adequately. Afterwards, 200 μL chloroform was added to the lysate, and the mix was shaken vigorously for 15 s and spun at 12 000 rpm, 4°C for 15 min. The supernatant was transferred to a fresh tube with 500 μL isopropanol, mixed completely, and centrifuged for 15 min. The RNA was precipitated with 1000 μL 75% cold ethanol and collected by centrifugation (10 000 rpm, 5 min). At the end of the extraction, RNA pellets were suspended in 100 μL of Rnase-free water.

#### cDNA synthesis

The reverse transcription reaction mixtures contained 2 μg of RNA samples that were treated with RNase-free DNase (Roche), Oligo(dT)_18_ 1 μL, 5× reaction buffer 4 μL, Ribolock^™^ RNase inhibitor (1μl), 10 mM dNTP Mix 2μL, 200 U/μL reverse transcriptase 1 μL, and diethylpyrocarbonate water to a final volume of 20 μL. The reaction mixtures were incubated at 65°C for 5 min. Then, reverse transcriptase was inactivated by incubation at 42°C for 60 min, then 70°C for 5 min, then cooled at 4°C and stored at -20°C. Finally, cDNA was generated using a RevertAid^™^ First Strand cDNA Synthesis Kit (Fermentas; Thermo Fisher Scientific).

#### Primers

The primers used to amplify the 82 bp *agrA* sequence were 5’- ACG TGG CAG TAA TTC AGT GTA TGT T -3’ (forward), and 5’- GGC AAT GAG TCT GTG AGA TTT TGT -3’ (reverse). For the 250 bp *sarA* sequence, they were 5’-GCT GTA TTG ACA TAC ATC AGC GAA A-3’ (forward), and 5’-CGT TGT TTG CTT CAG TGA TTC GT-3’ (reverse). For the 53 bp RNAIII sequence, they were 5’-GAA TTT GTT CAC TGT GTC GAT AAT CCA TTT-3’ (forward), and 5’-GAA GGA GTG ATT TCA ATG GCA CAA GAT AT-3’ (reverse). For the 86 bp *ica* sequence, they were 5’-TCG CAC TCT TTA TTG ATA GTC GCT ACG AG-3’ (forward) and 5’-TGC GAC AAG AAC TAC TGC TGC GTT AAT-3’ (reverse). For the 83 bp 16S rRNA sequence, they were 5’-CCA TAA AGT TGT TCT CAG TT -3’ (forward) and 5’-CAT GTC GAT CTA CGA TTA CT-3’ (reverse). For the 251 bp *gyrA* sequence, they were 5’-TGG CCC AAG ACT TTA GTT ATC GTT ATC C-3’ (forward) and 5’-TGG GGA GGA ATA TTT GTA GCC ATA CCT AC-3’ (reverse). For the 153 bp *gmk* sequence, they were 5’- TCG TTT TAT CAG GAC CAT CTG GAG TAG GTA-3’ (forward) and 5’-CAT CTT TAA TTA AAG CTT CAA ACG CAT CCC-3’ (reverse).

#### RTQ-PCR

Polymerase chain reactions were performed in total volume of 20 μL containing 12.5 μL of SYBR Premix Ex Taq (ROX), 0.5 μL each of forward and reverse primer, 1 μL of cDNA, 10.5 μL of ddH_2_O (ROX), as recommended by the manufacturer. We quantified cDNA using a StepOne Plus Real-Time PCR System (Applied Biosystems, USA) with FastStart Universal SYBR Green Master (ROX). Cycling parameters were as follows: holding stage of 95°C for 10 min; 40 cycles at cycling stage; 95°C for 15 s, 60°C for 60 s, and one melt curve stage of 95°C for 15 s, then 60°C for 60 s, and 95°C for 15 s. All sample experiments were performed in duplicate and analyzed in triplicate; and 16S rRNA, *gyrA*, and *gmk* were used as internal housekeeping control genes to normalize the expression levels between samples in parallel. The RTQ-PCR data were analyzed using the relative quantitative (2^**-ΔΔCt**^) method described in the Applied Biosystems User Bulletin No.2. The experiments were performed three times in parallel regardless of whether they were normalized by 16S rRNA, *gyrA*, *or gmk*.

### Statistical analysis

We present the quantitative culture results and relative expressions of genes from all groups as means ± S.D. Statistical comparisons between groups were made using analysis of variance on the log-transformed data with the significant difference test. Comparisons between any two means were achieved using additional post-hoc statistical tests, ang significance was accepted when the P value was < 0.05.

## Results

### The MICs of antimicrobial agents and effects of baicalein on bacterial growth *in vitro*

The MICs of baicalein, CLR, and VCM for the *S*. *aureus* strain 17546 used in the study were 256 μg/mL, 128 μg/mL and 1 μg/mL respectively. The MICs of baicalein, CLR, and VCM for ATCC 29213 (used for quality control) were 256 μg/mL, 2 μg/mL and 0.5 μg/mL, respectively. Clarithromycin was used as a positive control.

**Fig 1:**
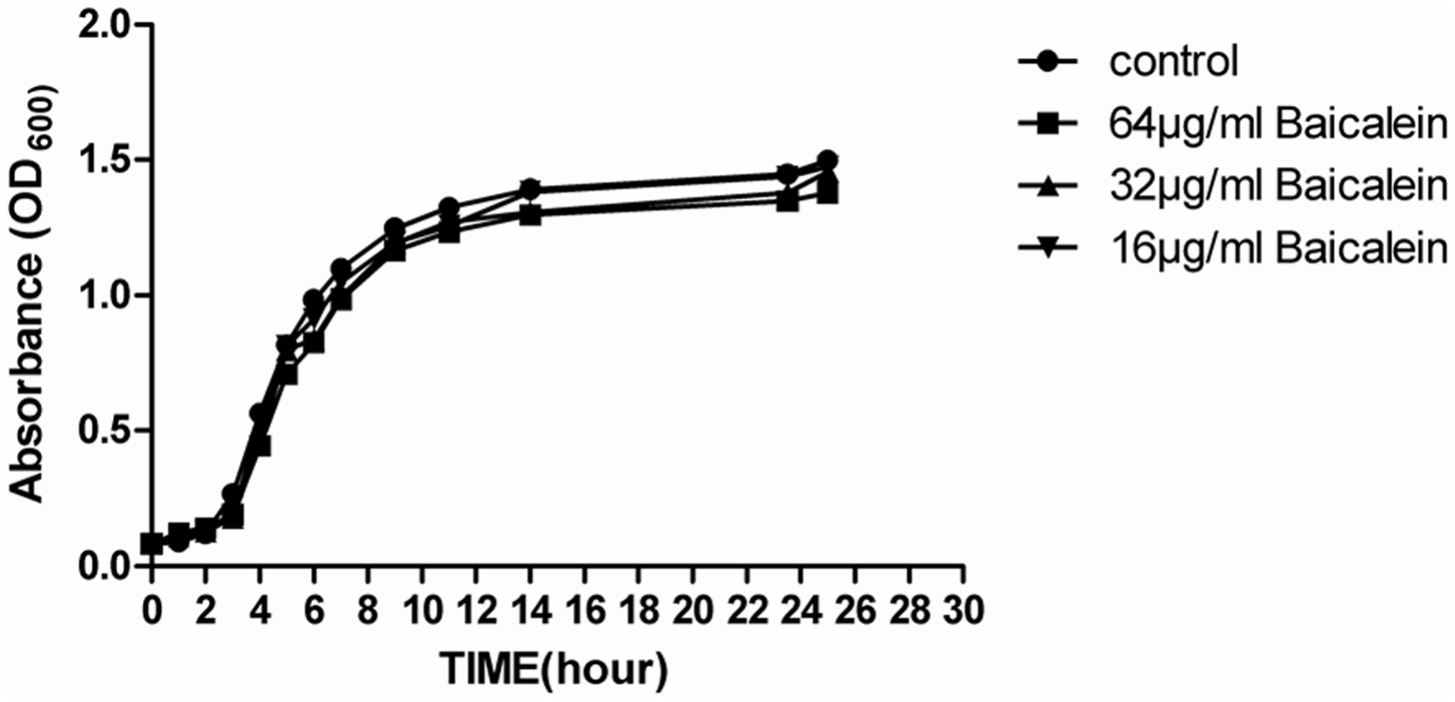
Growth curves of *S*. *aureus* at sub-inhibitory concentrations of baicalein. Baicalein at 16, 32 and 64 μg/mL had no effect on bacterial growth.

To test whether the sub-inhibitory concentration of baicalein had antibacterial activity, 1000 CFUs of *S*. *aureus* were grown for 26 h in 16, 32, and 64 μg/mL of baicalein (final concentrations). As shown in Fig 1, the sub-inhibitory concentrations of baicalein had no effect on bacterial growth.

**Fig 2:**
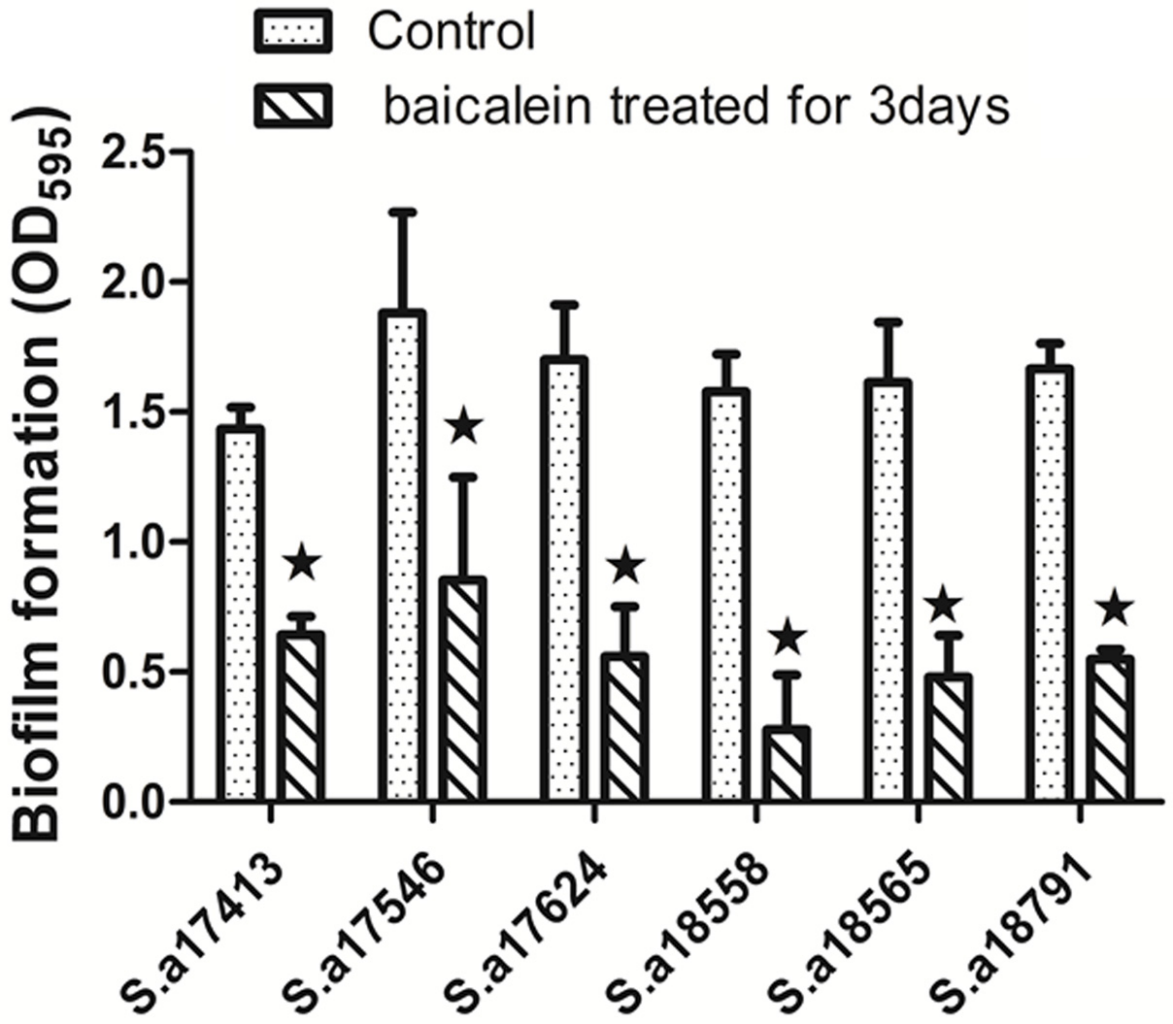
Inhibition of biofilm formation in clinical *S*. *aureus* strains by baicalein. The biofilms were stained with crystal violet and detected by OD_595_ (shown as the average of 3 experiments).

To study whether baicalein inhibits biofilm formation, bacterial suspensions of *S*. *aureus* strains 17413, 17546, 17624, 18558, 18565, and 18791 were treated with 32 μg/mL baicalein for 3 days (Fig 2). The biofilms were then stained with crystal violet and absorbance detected by OD_595_. Results showed that baicalein inhibited biofilm formation in our clinical *S*. *aureus* strains.

### Effect of baicalein on *S*. *aureus* biofilm formation *in vitro*

**Fig 3:**
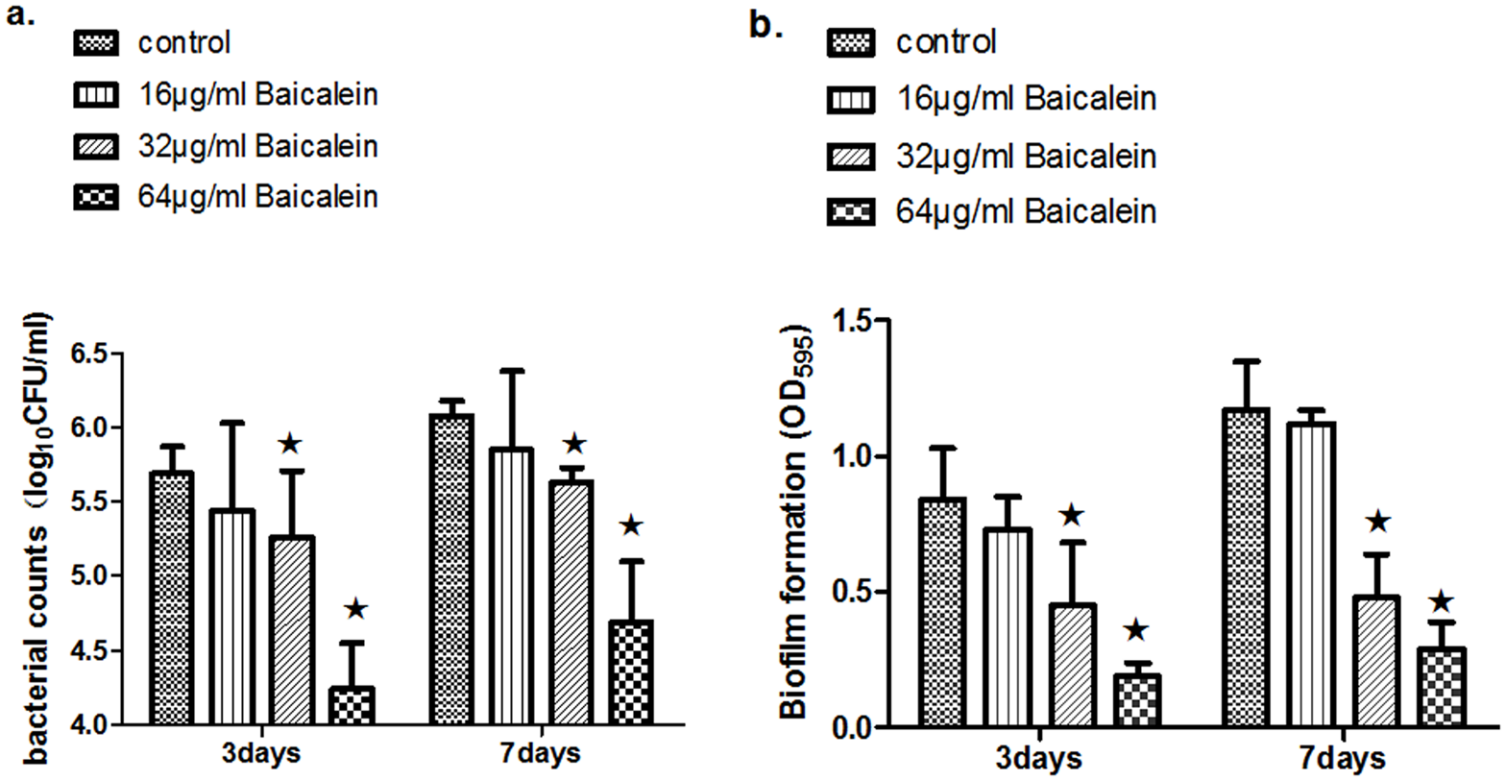
**(a) Biofilm bacterial counts.** Three- and 7-day day biofilm bacterial counts were obtained after exposure to 16 μg/mL, 32 μg/mL, and 64 μg/mL baicalein. (★ compared to control group, P< 0.05, figure shown as the average of 3 experiments). **(b) Crystal violet assay.** Crystal violet assay assessed the biomass after exposure to 16 μg/mL, 32 μg/mL, and 64 μg/ml baicalein. (★ compared to control group, P< 0.05, figure shown as average of 3 experiments).

**Fig 4:**
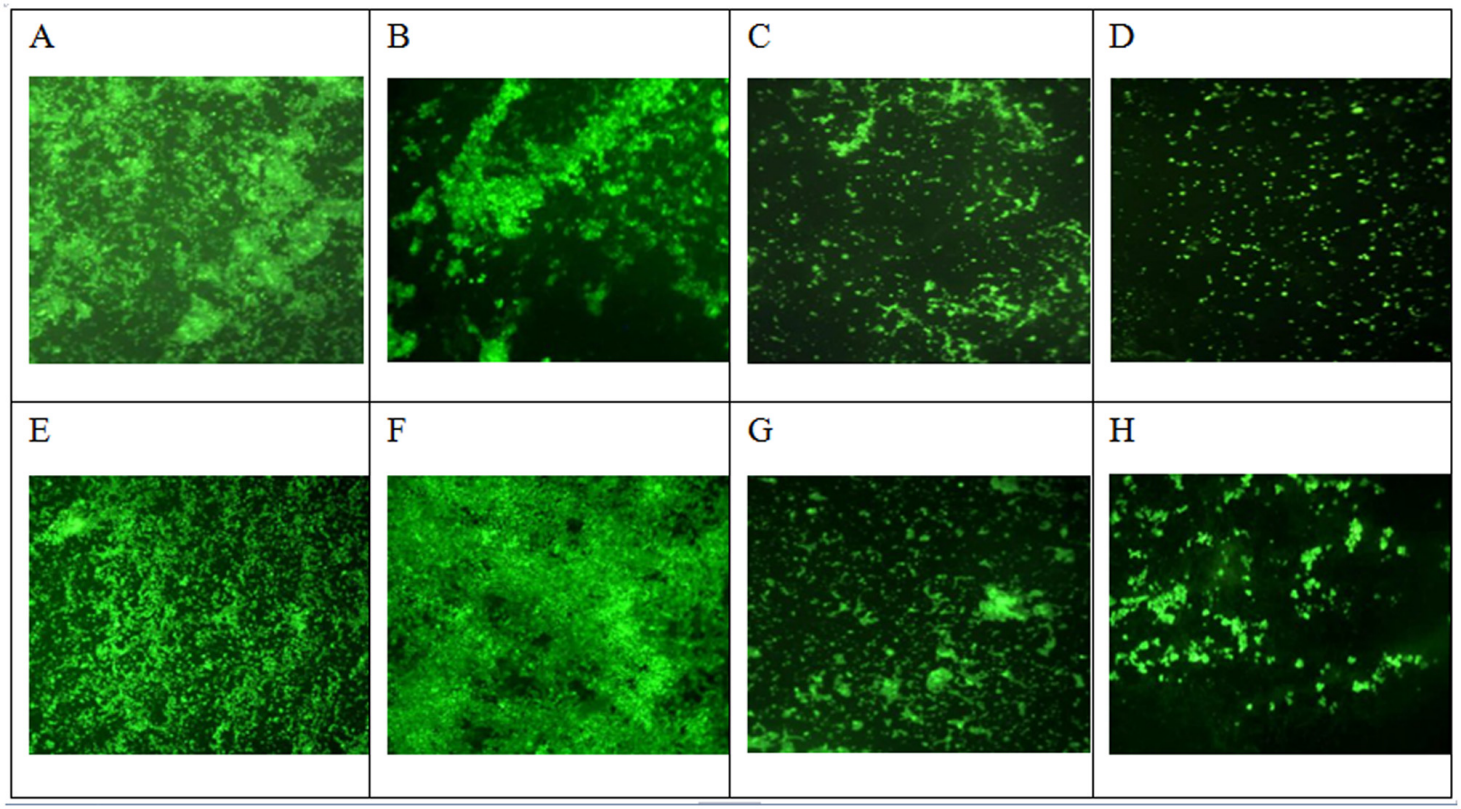
Impact of baicalein as assessed by fluorescence microscopy(200×). Static biofilms after exposure to baicalein for 3 days (top) and 7 days (bottom) were assessed by SYTO9. *S*. *aureus* within biofilms on polystyrene slices diplay green fluorescence. Controls group (A, E); 16 μg/ml baicalein group(B, F); 32 μg/ml baicalein group (C,G); 64 μg/ml baicalein group (D,H) A, E: control group; B, F: 16 μg/mL baicalein group; C, G: 32 μg/mL baicalein group; D, H: 64 μg/mL baicalein group.

To test for the effects of baicalein on biofilm formation *in vitro*, *S*. *aureus* cells were incubated with 16, 32, and 64 μg/mL of baicalein in polystyrene plates for 3 and 7 days at 37°C. The biofilms were stained with crystal violet, and OD was determined. We found that baicalein reduced cell attachment (Fig 3a) and biomass (Fig 3b) in a concentration-dependent manner regardless of whether the cultures were grown for 3 or 7 days. Baicalein demonstrated activity against *S*. *aureus* biofilms at 32 and 64 μg/mL, but 16 μg/ml baicalein had no effect. Moreover, fluorescence microscopy revealed that both 32 and 64 μg/mL baicalein inhibited *S*. *aureus* biofilm formation, showing bacterial counts that were lower than the control, while the 16 μg/mL baicalein had almost no effect (Fig 4).

### Combined efficacy of baicalein plus VCM

**Fig 5:**
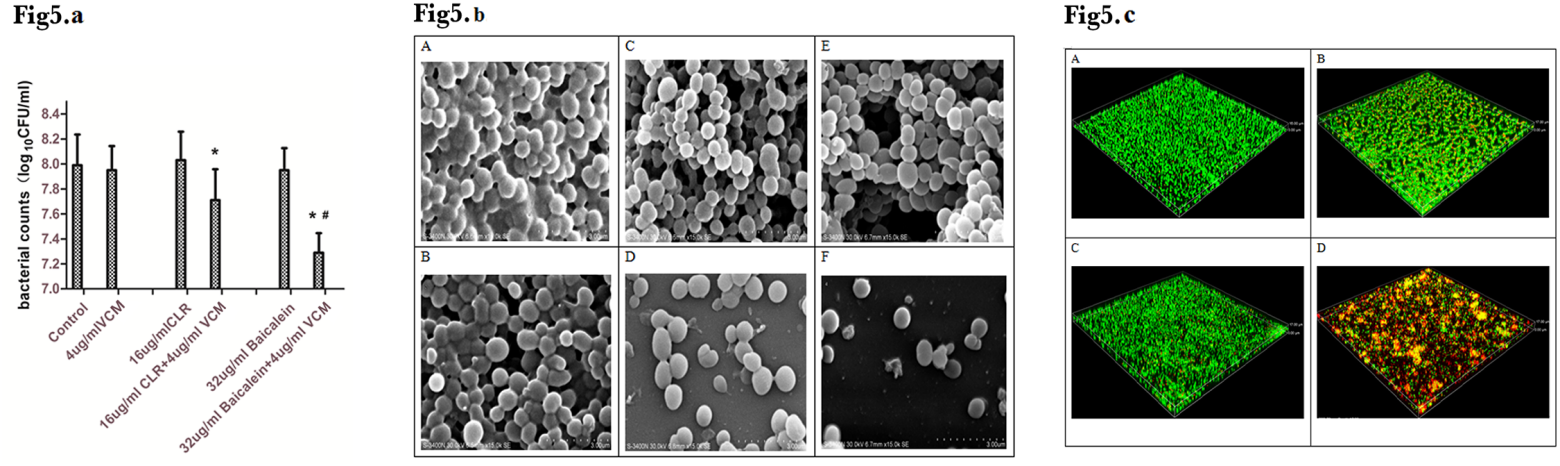
**(a) Use of baicalein as adjunct therapy with conventional antibiotics for 3-day biofilms.** Biofilms were formed on polystyrene carriers for 3 days by growing of *S*. *aureus* in TSB-G, then treating with VCM alone or combination with baicalein or CLR for 12 h (* compared to the control group, P < 0.001; # compared to the CLR+VCM group, P < 0.05; average of 3 experiments). **(b) Three-day biofilm scanning by SEM (×15000)** A: control group; B: 4 μg/mL VCM; C: 16 μg/mL CLR; D: 16 μg/mL CLR + 4 μg/mL VCM; E: 32 μg/mL baicalein; F: 32 μg/mL baicalein + 4 μg/mL VCM. **(c) Three-day biofilm scanning by CLSM (×400)** A: control group; B: 4 μg/mL VCM C: 32 μg/mL baicalein; D: 32 μg/mL baicalein + 4 μg/mL VCM.

**Fig 6:**
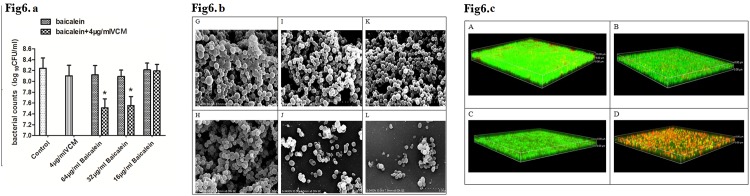
**(a) Viable bacterial counts of 7-day biofilms after reaction with medicaments for 12 h.** Biofilms were formed on polystyrene carriers for 7 days by growing *S*. *aureus* in TSB-G, then treating with VCM alone or combination with 16 μg/mL, 32 μg/mL, and 64 μg/mL baicalein for 12 h(* compared to the control group, P < 0.001, average of 3 experiments). **(b) 7 day BF scanning by SEM (×8000)** G: control group; H: 4 μg/mL VCM; I: 16 μg/mL CLR; J: 16 μg/ml CLR + 4 μg/ml VCM; K: 32 μg/mL baicalein; L: 32 μg/mL baicalein + 4 μg/mL VCM. **(c) Seven-day BF scanning by CLSM (×400)** A: control group; B: 4 μg/mL VCM; C: 32 μg/mL baicalein; D: 32 μg/mL baicalein + 4 μg/mL VCM.

Clarithromycin has been used in previous *S*. *aureus* anti-biofilm studies [5]. Here, we compared it to baicalein to determine whether baicalein was more effective in disrupting *S*. *aureus* biofilms than macrolide antibiotics. Three-day biofilms formed on polystyrene slices were treated with 16 μg/mL (⅛ MIC) of CLR or 16 μg/mL (⅛ MIC) of baicalein, with or without VCM. Seven- day biofilms were treated with serial dilutions of baicalein with or without VCM. Although CLR, baicalein, and VCM alone did not eradicate *S*. *aureus* in the 3-day biofilms, treatment with CLR+VCM or baicalein+VCM remarkably reduced the number of bacteria on the carrier. Furthermore, the bacterial counts on biofilms treated with baicalein+VCM were lower than those treated with CLR+VCM (Fig 5a). Viable bacterial counts on the 7-day biofilms did not decrease after treatments with 64, 32 or 16μg/mL of baicalein, nor did they decrease with 16μg/mL treatment of baicalein+VCM. However, VCM combined with either 32μg/mL or 64μg/mL baicalein did reduce bacterial counts (Fig 6a).

**Fig 7:**
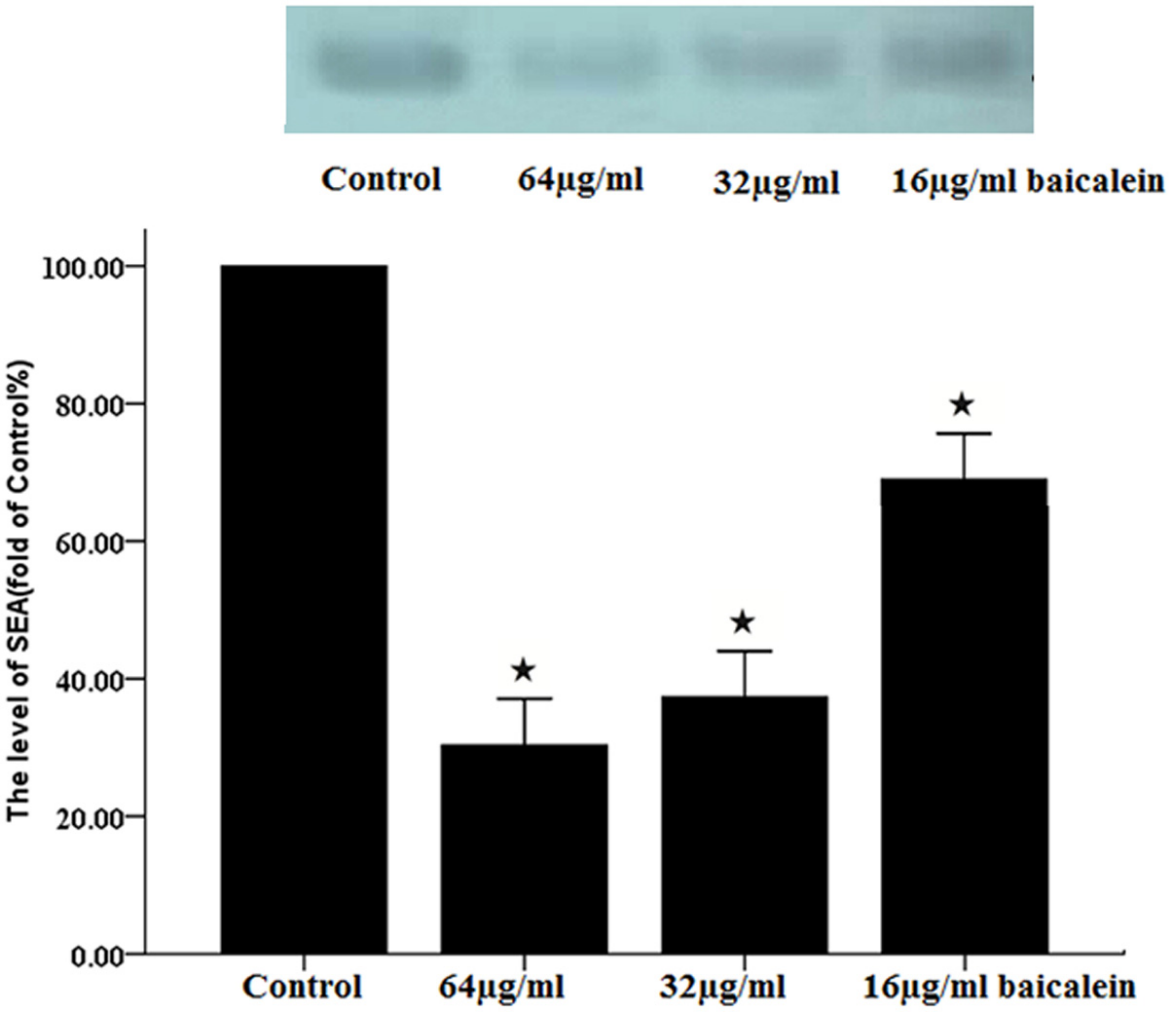
SEA detected by western blot. Upper: Western blot analysis of SEA from control group and groups treated with 64, 32, and 16 μg/ml baicalein for 5 h. Lower: Band intensities were quantitated by densitometry. Each lane was divided by the logarithmic number of cells in order to evaluate the amount of SEA per cell. (* P < 0.05, compared to the control group with one-way ANOVA). Data shown are the average of 3 experiments.

Scanning electron microscopy was conducted on on 3-day (Fig 5b) and 7-day (Fig 6b) *S*. *aureus* biofilms that were treated with VCM, baicalein+ VCM, and CLR+VCM. Evaluation by SEM showed that treatment with baicalein or CLR reduced the extracellular matrix. Baicalein, CLR, and VCM alone did not eradicate *S*. *aureus* bacteria within the formed biofilms. However, combination treatment with CLR+VCM or baicalein+VCM proved bactericidal, with baicalein+VCM being more effective than CLR+VCM.

The 3-day (Fig 5c) and 7-day (Fig 6c) biofilms treated with either VCM or baicalein+VCM were observed by CLSM. Results showed that VCM could barely penetrate the biofilm to kill bacteria. The VCM group had almost green fluorescence(representing viable cells), the same as the control group. The sub-inhibitory concentration of baicalein also had no effect on *S*. *aureus* and showed almost green fluorescence, while the baicalein+VCM group had almost red fluorescence (representing dead cells).

### Virulence factor detection

#### SEA

Western blot was used after baicalein treatment to determine whether baicalein affects SEA level. As shown at the top of Fig 7, there are four bands representing the amount of SEA after baicalein-treatment (0, 16, 32 and 64 μg/mL). After 5 hours of treatment with 32 or 64 μg/mL baicalein, the SEA band was lighter that of the control. The amounts of SEA/Iog_10_(CFU) of the 64, 32 and 16μg/mL baicalein groups dramatically lower than that of the control. (Fig 7)

#### Hla

**Fig 8:**
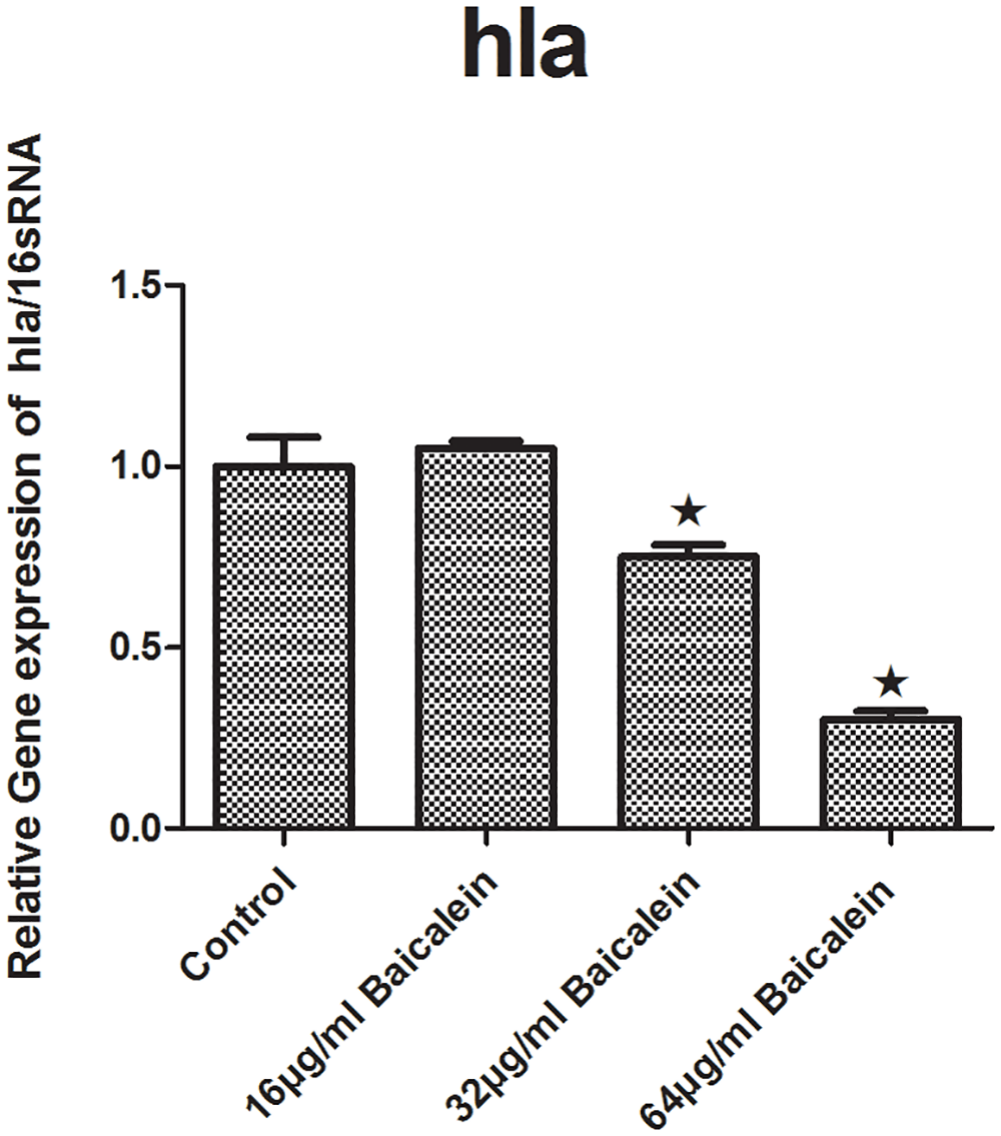
Relative expression of the *hla* (using 16sRNA as the internal parametric gene). Comparative measurement of the relative expression shown as average of 3 experiments. (★ compared to the control group, P < 0.001).

We performed RTQ-PCR to determine whether baicalein can affect the production of *hla*. After treatment of the bacterial suspensions with 16, 32 or 64 μg/mL baicalein for 5 h, the relative gene expression levels of *hla*/16S RNA were calculated. We found that the gene expression levels significantly differ between the 32 μg/mL and 64 μg/mL baicalein groups and the control group (Fig 8).

#### Intervention to the QS system

**Fig 9:**
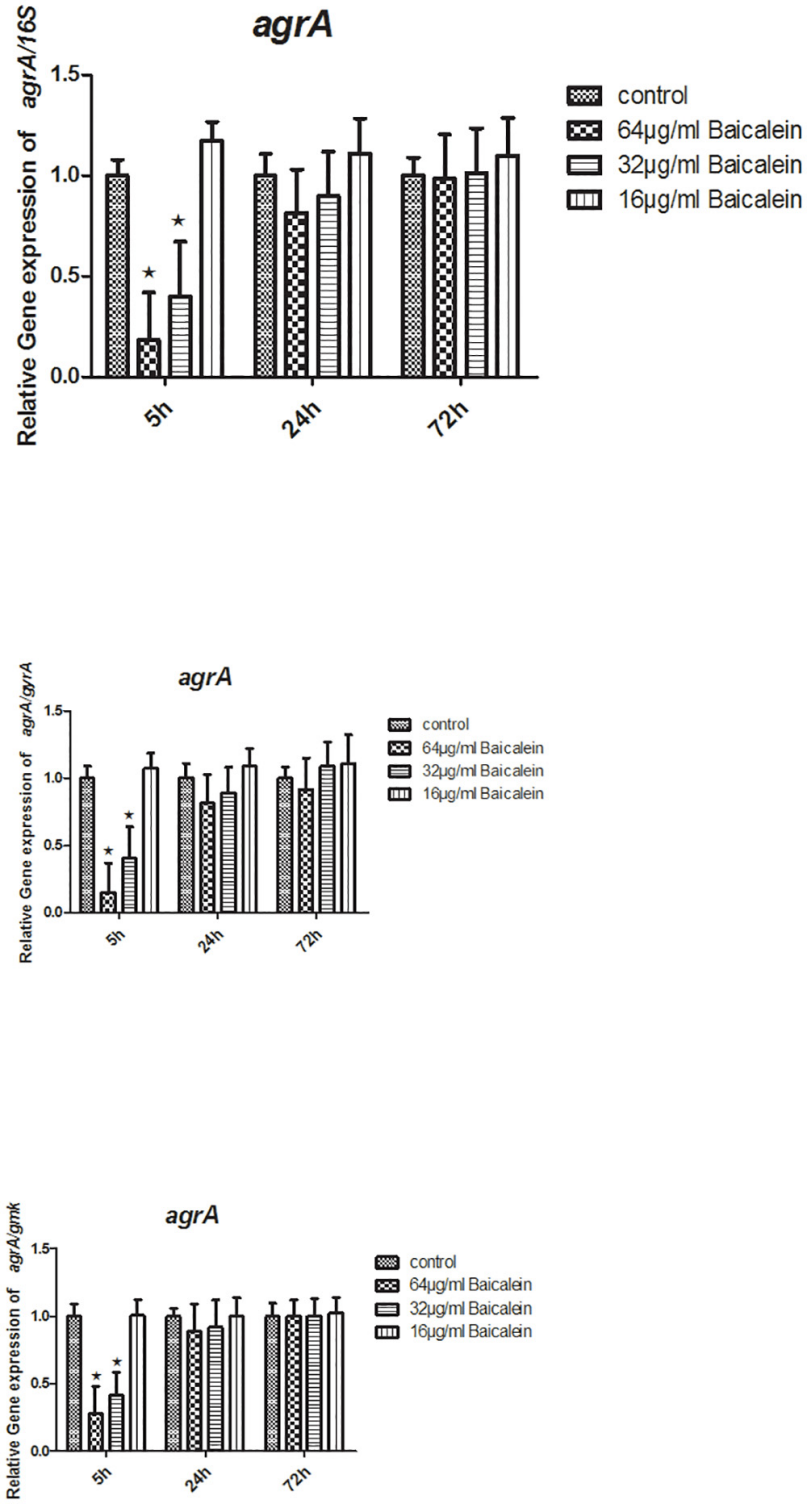
Relative expression of the *agrA* (using 16S rRNA / *gyrA* / *gmk* as the internal parametric gene). Comparative measurement of the relative expression shown as average of 3 experiments. (★ compared to the control group, P < 0.05).

**Fig 10:**
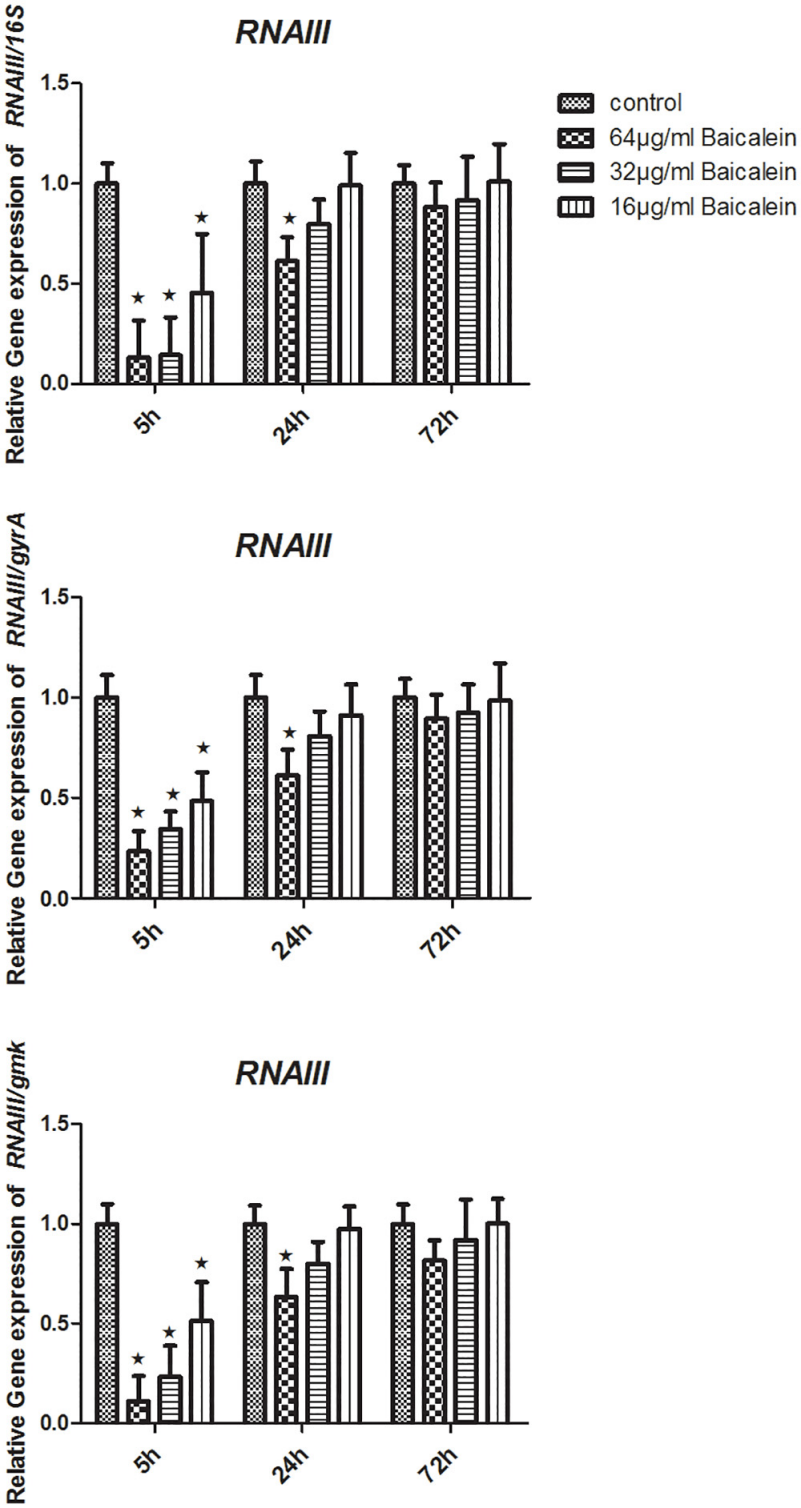
Relative expression of the RNAIII (using 16S rRNA / *gyrA* / *gmk* as the internal parametric gene). Comparative measurement of relative expression shown as an average of 3 experiments (★ compared to the control group, P < 0.05).

**Fig 11:**
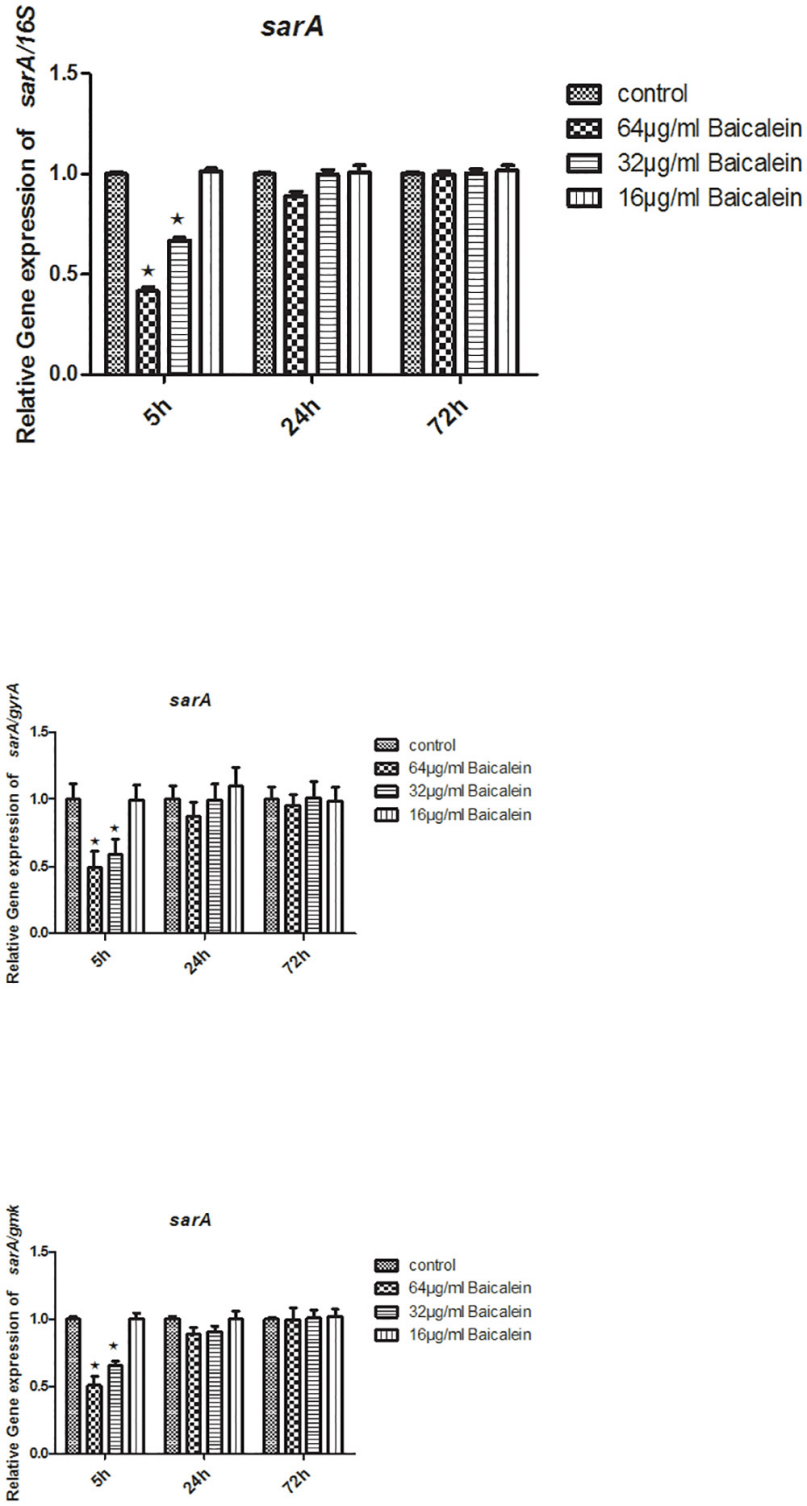
Relative expression of the *sarA* (using 16S rRNA / *gyrA* / *gmk* as the internal parametric gene). Comparative measurement of relative expression shown as an average of 3 experiments (★ compared to the control group, P < 0.05).

**Fig 12:**
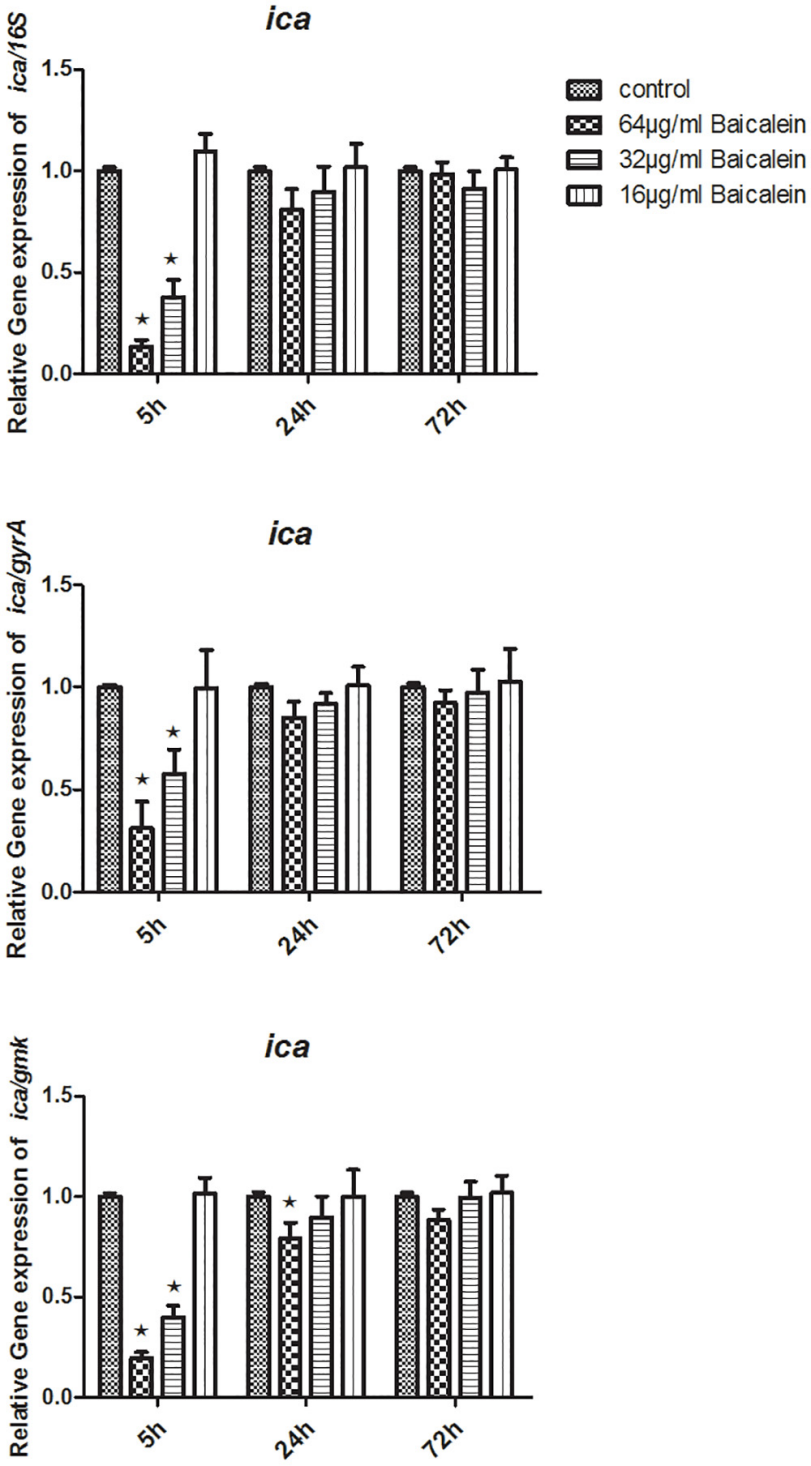
Relative expression of the *ica*(using 16S rRNA / *gyrA* / *gmk* as the internal parametric gene). Comparative measurement of relative expression shown as an average of 3 experiments (★ compared to the control group, P < 0.05).

To measure the levels of specific RNA transcripts in different samples, total RNA extracts were used as a template for cDNA synthesis. Then, 2 μg of cDNA copies were tested using RTQ-PCR. Relative quantitative values were calculated using the 2^**-ΔΔCt**^. Here, the 16S rRNA, *gyrA*, and *gmk* were used as internal housekeeping control genes. After the bacterial suspensions were treated with either 32 or 64 μg/mL baicalein for 5 h, the transcription levels of *agrA*, *sarA*, and *ica* statistically declined (P<0.05), regardless of whether they were normalized by 16S rRNA, *gyrA*, or *gmk*. Treating the strains with 16 μg/mL baicalein (5 h), or 32 μg/mL baicalein (24 h or 72 h), or 64 μg/mL baicalein (24 h or 72 h) did not change *agrA*, *sarA*, and *ica* expression levels. Treatment with 64, 32 or 16 μg/mL baicalein for 5 h or 64 μg/mL baicalein for 24 h statistically lowered the RNAIII expression levels (P < 0.05). No significant difference in RNAIII expression levels resulted from treatment with 32 or 16 μg/mL baicalein for 24 h, nor was there a difference after 72 h when compared to that of the control (Figs 9–12).

## Discussion

In a previous study, we demonstrated that Radix Scutellariae decoctions have synergistic bactericidal effects with antibiotics, which prevents biofilm formation [9]. This time, we limited the study to the active ingredient baicalein and a specific strain of *S*. *aureus* (17546). Baicalein (5,6,7-trihydroxyflavone) is an aglycone of baicalin, a flavone isolated from the roots of *Scutellaria baicalensis*. Baicalein also found in *Oroxylum indicum* (Indian trumpet flower). The flavonoid has been shown to inhibit certain types of lipoxygenases [10] and act as an anti-inflammatory agent [11]. http://en.wikipedia.org/wiki/Baicalein-cite_note-2#cite_note-2It inhibits endothelin-1 induced pulmonary artery smooth muscle cell proliferation by preventing expression of transient receptor potential canonical 1 channels [12]. Research on animal models indicates baicalein has possible anti-depressant effects [13]. It also inhibits of cytochrome P450 2C9 [14], an enzyme that metabolizes drugs in the body [15]. Most notably, baicalein has demonstrated antimicrobial effects against *Dysentery bacillus*, *Corynebacterium diphtheriae*, *Pseudomonas aeruginosa*, *Staphylococcus aureus*, *Streptococcus*, *Pneumonic diplococcus*, and *Meningococcus*.

In this study, baicalein’s anti-biofilm properties were assessed phenotypically and genetically. The results confirmed that baicalein was effective at preventing *S*. *aureus* biofilm formation even at sub-inhibitory concentrations. While baicalein and VCM individually had no effect on bacterial counts in established biofilms, the combination of the two caused a dramatic effect on dispersal. Therefore, combining baicalein with antibiotics is a possible novel method for prophylaxis and treatment of biofilm-associated infections.

We found that baicalein inhibited biofilm formation of clinically isolated strains of *S*. *aureus* (17413, 17546, 17624, 18558, 18565 and 18791). However, *S*. *aureus* 17546 was used for the remainder of the study, because it had the greatest ability to form biofilms.

A few studies [16,17] have proven that many TCMs play a role in preventing biofilm formation. However, this is the first study to investigate the effect of baicalein on *S*. *aureus* biofilm. In this work, 64, 32, or 16 μg/mL baicalein and DMSO (0.8%, 0.4%, and 0.2%, respectively) were not bactericidal or bacteriostatic against *S*. *aureus*. However, bacterial count and fluorescence microscopic assay revealed that treatment with 64 and 32μg/mL baicalein reduced the number of bacteria within the biofilms, and SEM demonstrated that 64 and 32μg/mL baicalein reduced the biomass. At 16 μg/mL, baicalein lowered neither the bacterial count nor the biomass, regardless of a 3- or 7-day incubation period. Therefore, we believe that baicalein is effective at preventing *S*. *aureus* biofilm formation in a dose-dependent manner.

Clinically, many biofilm-associated infections occur without prevention. In our work, baicalein disrupted the already formed biofilms to increase antibiotic permeability. To some extent, this does make clinical sense. Fujimura [5] have shown that *S*. *aureus* biofilms are not eradicated by CLR or VCM alone. It is treatment with CLR+VCM that destroys *S*. *aureus* biofilms formed on medical devices to eradicate *S*. *aureus*. In our study, we used 16 μg/mL CLR (⅛ MIC) as a positive control to assess the combined effects of 32 μg/mL baicalein (⅛ MIC) with VCM on biofilms that were established for 3 days.

Our assays showed that either baicalein or CLR could destroy biofilms. In fact, baicalein was able to eradicate 7-day biofilms in a dose-dependent manner (Fig 6). When visualized by SEM and CLSM, baicalein eradicated extracellular matrices and baicalein+VCM eliminated sessile *S*. *aureus* cells from within the biofilms.

In our previous work, the fractional inhibitory concentration of baicalein and VCM was 2, confirming that baicalein in combination with VCM has neither synergistic nor antagonistic effects on MIC. However, although *S*. *aureus* could not be eradicated by VCM alone, both baicalein+VCM and CLR+VCM could destroy *S*. *aureus*. More notably, baicalein+VCM was more effective than CLR+VCM (Fig 5). We conclude that sub-inhibitory concentrations of baicalein could destroy biofilms *in vitro* and eradicate *S*. *aureus* when combined with VCM, and this combined effect was stronger than CLR alone. Presumably, baicalein may be a preferred treatment for biofilm infections, by either inhibiting biofilm formation or through its bactericidal effect as adjunct with VCM.

Biofilm formation is thought to require adhesion of cells to a solid substrate, which creates multiple layers of cells. Intercellular adhesion requires PIA, which can be synthesized by products of the intercellular adhesion (*ica*) locus. Deleting the *ica* locus disrupts production of PIA [18,19] and the formation of biofilms, making it another potential therapeutic target.

The QS system controls *S*. *aureus* biofilm formation and release of virulence factors [20,21]. Researchers believe that the accessory gene regulator (*agr*) system regulates the QS in staphylococci [22]. In fact, *agr* plays a pivotal role in regulating virulence factor expression [23], making it a potential therapeutic target [24].

The RNAIII activating peptide is thought to be a type of auto inducing peptide [25,26] that can phosphorylate its target molecule to activate the *agr* system, which increases production of auto inducing peptides and AgrC to enhance the adhesion of the bacteria [27,28]. Recent findings [19,29] indicate that RNAIII is a regulatory mRNA molecule that not only regulates biofilm formation but also induces toxin production, such as enterotoxin, plasma-coagulase, hemolysin and thermostable nuclease. In addition, it is a member of the staphylococcal accessory regulator A (*sarA*) family of transcriptional regulators [30]. Recent evidence indicates that SarA, a central regulatory element that controls the production of *S*. *aureus* virulence factors, is essential for the synthesis of polysaccharide intercellular adhesin (PIA) [31].

We have shown that baicalein could inhibit biofilm formation by *S*. *aureus* phenotypically. To determine whether baicalein inhibits the QS system and virulence, we measured the transcriptional level of *ica*, *agrA*, RNAIII, *sarA*, and *hla* through RTQ-PCR and monitored the production of SEA through western blot analysis. The genes and virulence factors were observed after 64, 32, and 16 μg/mL baicalein were added to the *S*. *aureus* suspensions for 5 h, 24 h, and 72 h. The RTQ-PCR data were analyzed by the relative quantitative (2^**-ΔΔCt**^) method. At the same time, we monitored both the target gene and the housekeeping genes(16S rRNA, *gyrA*, *gmk*) for every group. Then, the expression difference of the target gene and any one housekeeping gene was concurrently compared to the control group. Sabersheikh et al [8] showed a relatively constant level of 16S rRNA between 2 and 7 h following inoculation, so, we analyzed the samples after 5 hours of baicalein exposure. We found that 64 and 32 μg/mL of baicalein could down-regulate the expressions of *ica*, *agrA*, RNAIII, *sarA*, and *hla*, as compared with that of the control group, while 16 μg/mL baicalein proved ineffective against all except RNAIII. Thus, sub-inhibitory concentrations of baicalein effectively inhibited the QS system of *S*. *aureus* in a dose-dependent manner, resulting in inhibition of biofilm formation. When the incubation time was prolonged (24 h or 72 h), 64 and 32 μg/mL of baicalein had no effect on expression. We inferred that the QS system was active at about 5 hours after incubation, at which time baicalein showed strong inhibition at the genetic level. This may guide us to time drug treatment for optimal biofilm inhibition.

To determine the levels of SEA, we made the same volume of bacterial suspension for the control and baicalein-treated groups. After incubation with baicalein or not for 5 h, we took 100 μL of suspension from each group for centrifugation and for CFU counting. The SEA production was divided by the cell logarithmic number to evaluate the amount of SEA per cell. The amount of SEA per cell significantly decreased upon exposure to 64, 32, or 16 μg/mL of baicalein.

Biofilm formation is a dynamic process—from adherence to formation, maturity to dispersion—all controlled by a variety of genes. Thus, the mechanisms for prevention and eradication of biofilms differ. Perhaps baicalein competitively inhibits the QS signaling molecule AgrD when combined with AgrC, or it might block RNAIII activating peptide when combined with the target of RNAIII activating protein. This would block the phosphorylation of the downstream cascade pathway, inhibiting the expression of the corresponding gene to prevent biofilm formation. Baicalein might be considered a QS inhibitor because of its ability to block the cell-to-cell signal transduction that is regulated by the QS system, and thus inhibiting expression of QS-related genes. The specific mechanism responsible for inhibiting the QS system requires further study and discussion, but the net outcome of QS inhibition is inhibition of biofilm formation and retention of bacteria in a planktonic state.

To conclude, baicalein destroyed biofilms to enhance antibiotic permeability, effectively inhibit the *S*. *aureus* QS system to biofilm formation. Therefore, we believe baicalein is potential novel treatment against *S*. *aureus* biofilm-related infections.

The results remain to be confirmed by a large number of preclinical *in vivo* animal experiments and clinical trials to test the effectiveness, toxicity, and pharmacokinetics in humans. Our future research will focus on animal experiments *in vivo* to identify the precise mechanism by which baicalein inhibits *S*. *aureus* biofilm formation.
